# Comparison of DLP and CTDI in somatom Definition Flash and GE revolution computed tomography scanners at AIIMS, India

**DOI:** 10.6026/973206300221712

**Published:** 2026-03-31

**Authors:** Lalit Gupta, Ashok Sharma, Ashish Kumar Shukla, Sumit Tyagi

**Affiliations:** 1 Department of Radiodiagnosis and Imaging Technology, Santosh Deemed to be University Ghaziabad, Uttar Pradesh, India

**Keywords:** Computed tomography, dose length product, diagnostic reference levels, radiation dose, tomography dose Index

## Abstract

The increasing radiation exposure from computed tomography (CT) scans raises concerns about patient safety, necessitating the comparison of radiation dose parameters across different CT scanners. This study compares the Dose Length Product (DLP) and Computed Tomography Dose Index (CTDI) values between the Somatom Definition Flash and GE Revolution CT scanners at AIIMS, New Delhi. Our findings reveal that the Somatom scanner consistently operates within international Diagnostic Reference Levels (DRLs), while the GE scanner shows significantly higher radiation doses, particularly for chest and abdomen scans. This research advances the understanding of CT dose optimization and highlights the need for scanner-specific protocols to ensure radiation safety.

## Background:

Computed Tomography (CT) has become an important diagnostic tool in modern medicine, offering rapid and high-resolution imaging capabilities [1]. However, the increasing use of Computed Tomography has increase significant concerns regarding patient radiation exposure, particularly given the associated risk of stochastic effects such as radiation-induced malignancies [2]. To avoid these risks and promote dose optimization, various international regulatory bodies and professional organizations have established Diagnostic Reference Levels (DRLs) as a means to guide appropriate radiation use [3]. The European Commission (EC) [4] and the United Kingdom (UK) [5] have developed well-known DRLs for commonly performed Computed Tomography examinations, including scans of the head, chest and abdomen [6]. These DRLs serve as benchmarks for institutions to assess and refine their imaging protocols. In India, however, such benchmarking is still evolving and there is a need to compare local dose metrics with internationally recommended standards to ensure patient safety and quality assurance. This study aims to evaluate and compare radiation dose parameters specifically the Dose Length Product (DLP) and Computed Tomography Dose Index (CTDIvol) between two advanced multi-detector Computed Tomography systems, the Somatom Definition Flash (Siemens) and the GE Revolution (GE Healthcare), both currently in use at the All India Institute of Medical Sciences (AIIMS), New Delhi [7]. Therefore, it is of interest to compare the radiation dose parameters, Dose Length Product and CTDIvol, between the Somatom Definition Flash and GE Revolution Computed Tomography scanners at AIIMS, New Delhi, against international Diagnostic Reference Levels (DRLs).

## Methodology:

This study was done as a retrospective observation carried at the Department of Radiodiagnosis, All India Institute of Medical Sciences (AIIMS), New Delhi. Study was done with aim to assess radiation doses from routine Computed Tomography scans performed using two types of multi-detector Computed Tomography scanners which was the Somatom Definition Flash and the GE Revolution. Both non-contrast and contrast- enhanced studies of the head, chest and abdomen scans were included in the study. Data collection was done for six months, from January to June 2025. We used records from the hospital's digital imaging and reporting systems to identify all Computed Tomography scans done during this time for the three body regions. Only adult patients (18 years and older) scanned for regular diagnostic reasons (not emergencies) were considered. Scan which have a complete dose report with both CTDIvol and Dose Length Product values were considered for the study. Any scans with missing or incomplete data, images with major motion problems or repeat scans, as well as those from children or involving multiple phases of contrast, were not considered in the study. For each anatomical region head, chest and abdomen at least 50 scans per scanner type were reviewed to make the results reliable. The radiation dose parameters, CTDIvol (measured in mGy) and Dose-Length Product (DLP, in mGy.cm), were taken from auto-generated dose reports produced by the Computed Tomography scanners. Both scanners operated with standard hospital protocols designed for best image quality and diagnostic accuracy and the settings were matched as closely as possible. This included checking scan range, tube voltage, current control, pitch, rotation time and use of automatic exposure controls. After the data collection the recorded dose values were compared with safety standards set by both the European Commission (EC) and the UK National Diagnostic Reference Levels (NDRL) and other international DRL values. For analysis, the median values for each scanner and region were calculated. Differences between the scanners were tested using standard statistical methods considering p value <0.05 for statistical significance. The study followed all ethical requirements. Approval was granted by the Institutional Ethics Committee at AIIMS, with patient consent not required because the study used only previously recorded data.

## Results:

In this study, two major radiation dose metrics Dose-Length Product (DLP) and Computed Tomography Dose Index Volume (CTDIvol) were evaluated for routine adult CT scans performed on Somatom and GE scanners at AIIMS. The Somatom scanner produced DLP values well below both EC and UK DRLs for all anatomical regions, showing optimized scanning protocols. The GE scanner, in contrast, generated significantly higher DLPs for all regions, particularly in the chest (1020 mGy.cm) and abdomen (1150 mGy.cm), exceeding EC and UK reference levels. The excess dose reported for GE scans highlights the need for protocol review and dose optimization. Somatom values for CTDIvol stayed consistently below or near both UK and EC limits, confirming adequate dose management. GE values, however, showed elevated CTDIvol across all three regions, with the head (73 mGy) and chest (19 mGy) values higher than EC and UK limits. Abdomen CTDIvol (20 mGy) was within the broader EC range but remained above UK DRLs (10 mGy). These results emphasize the need for dose optimization protocols on GE systems, particularly for head and chest imaging, to minimize patient radiation exposure. Table 1 (see PDF) presents a comparison of Dose Length Product median values (in mGy.cm) recorded for Somatom and GE scanners with the European Commission (EC) and UK Dose Length Product reference levels. Table 2 (see PDF) compares the median CTDIvol values with UK NDRLs and EC guidelines for both scanners. Table 3 (see PDF) outlines the other state and international Computed Tomography Dose Index values (in mGy) across various countries and regions. Finally, Table 4 (see PDF) provides a comparison of other state and international Dose Length Product values (in mGy.cm) for different anatomical regions and scanners.

Somatom scanner consistently showed lower Dose Length Product values, showing better radiation dose optimization and safety. In contrast, the GE scanner demonstrated significantly higher Dose Length Product values across all regions, especially for Chest and Head CTs, showing a need for protocol review and optimization. When compared with the European Commission (EC) and IAEA reference levels, Somatom values were well within acceptable limits, whereas GE values often exceeded international diagnostic reference levels (DRLs), raising concerns regarding radiation exposure.

## Discussion:

This study aimed to evaluate radiation dose levels delivered during routine adult Computed Tomography scans at a tertiary care institution, using two different multidetector Computed Tomography scanners (Siemens Somatom and GE systems). Dose metrics, including Dose Length Product (DLP) and Computed Tomography Dose Index Volume (CTDIvol), were assessed for head, chest and abdomen Computed Tomography protocols. The Computed Tomography Dose Index (CTDI) values observed in our study using two different scanners Somatom and GE were compared with available data from other Indian states and international references. In head examinations, the Siemens Somatom scanner recorded a median Dose Length Product of 780 mGy.cm, which is below both the EC DRL (1050 mGy.cm) and the UK NDRL (960 mGy.cm), demonstrating appropriate dose optimization consistent with international standards. In contrast, the GE system showed a median head Dose Length Product of 1470 mGy.cm, exceeding both EC and UK thresholds, showing the possibility of unnecessary radiation exposure in routine protocols. Computed Tomography Dose Index vol values show a similar pattern. The Somatom measured 45 mGy, similar to the UK DRL (47 mGy) and EC DRL (60 mGy), whereas the GE system showed 73 mGy, exceeding both reference levels. Higher GE head values may be attributed to fixed high tube current, limited modulation controls, or non-optimized helical parameters. Comparable findings have been seen in international study by Atli *et al.* (2022), who showed head CTDIvol medians of 61.1 mGy on standard protocols in Turkey showing the need for optimization when scanners exceed DRLs [8]. In head CT, the Somatom scanner reported 45 mGy, which is comparable to values from South India (47 mGy) and IAEA CRPR (47 mGy) and lower than those from the UK (60 mGy), Ireland (58 mGy) and Canada (82 mGy). The GE scanner, however, showed a head Computed Tomography Dose Index of 73 mGy, which is notably higher than most regional and global references except Japan (85 mGy) and Canada (82 mGy). For head scans, the Somatom scanner had a Dose Length Product of 780 mGy.cm, which is below average, comparable to Turkey (810) and Syria (793) and lower than South India (1041), USA (962) and Canada (1302). The GE scanner recorded a very high Dose Length Product of 1470 mGy.cm, exceeding almost all reported values, including Japan (1350), Canada (1302) and Korea (1119.4). The performance gap is most pronounced in chest CT. The Somatom scanner achieved a very low median chest Dose Length Product of 217 mGy.cm, well beneath the EC (650 mGy.cm) and UK (560 mGy.cm) DRLs. Its CTDIvol value (6 mGy) is also below both guidelines (UK: 8.5 mGy; EC: 12 mGy), reflecting effective protocol optimization through techniques like automated exposure control and low-kVp imaging. However, the GE scanner yielded substantially elevated doses: CTDIvol of 19 mGy and Dose Length Product of 1020 mGy.cm, both nearly twofold above international reference levels. This mirrors trends reported in some multicenter European studies, where chest Computed Tomography DLPs ranged from 700-1100 mGy.cm depending on scanner calibration and user-dependent factors. These findings show that dose modulation and acquisition parameters in the GE system require immediate review to meet international safety standards. For chest CT, our Somatom scanner showed a Computed Tomography Dose Index of 6 mGy, which is lower than nearly all regional and international values, including Kerala (5 mGy), South India (10 mGy) and IAEA CRPR (9.5 mGy). The GE scanner showed a Computed Tomography Dose Index of 19 mGy, which is among the highest reported, surpassing those from Syria (22 mGy), Japan (15 mGy) and Canada (14 mGy). The Somatom scanner recorded a Dose Length Product of 217 mGy.cm, which is among the lowest values, even lower than Kerala (164) and Turkey (289). It shows effective dose optimization. The GE scanner, however, produced a very high Dose Length Product of 1020 mGy.cm, which is significantly higher than all national and international benchmarks, including UK (610), Japan (550) and EC (400). Similar trends were observed in abdominal Computed Tomography scans.

Somatom's CTDIvol of 7 mGy and Dose Length Product of 306 mGy.cm were again well below both EC (CTDIvol: 15 mGy; DLP: 780-900 mGy.cm) and UK limits (CTDIvol: 10 mGy; DLP: 700 mGy.cm). These are reflective of optimized scanning protocols and rational exposure justification. Conversely, the GE system had the highest abdomen Dose Length Product at 1150 mGy.cm and CTDIvol at 20 mGy. While the CTDIvol is within the broader EC range (often reported between 15-25 mGy), it still substantially exceeds UK standards, which dictate a CTDIvol of 10 mGy. Comparable values were noted by Sabiniewicz-Ziajka *et al.* where adult abdominal CTDIvols across multiple centers ranged from 12-20 mGy, identifying large variability attributable to scanner settings and user practices [9]. Similarly to our study, Sulieman *et al.* [10] found the mean and range of 28.2 (3.0-13.0) CTDIvol (in mGy) for the abdomen. For abdominal Computed Tomography scans, the Somatom scanner in our study reported the lowest Computed Tomography Dose Index of 7 mGy, which is significantly below the values reported from South India (12 mGy), Puducherry (16 mGy) and Kerala (9 mGy). Even when compared to international benchmarks such as the IAEA CRPR (10.9 mGy), Korea (10.58 mGy) and the UK (14 mGy), our Somatom Computed Tomography Dose Index remains lower. In contrast, the GE scanner recorded a higher abdominal Computed Tomography Dose Index of 20 mGy, which exceeds values from most regions except Syria (24.1 mGy) and falls within the range reported by the European Commission (13-35 mGy). The Somatom scanner in our study showed a relatively low Dose Length Product of 306 mGy.cm, which is lower than values reported from South India (550), Puducherry (482) and IAEA CRPR (696). It is also below international benchmarks such as the UK (910), Ireland (1120) and Korea (1511.41). In contrast, the GE scanner recorded a much higher Dose Length Product of 1150 mGy.cm, which is among the highest values, comparable to Ireland (1120) and slightly lower than Korea (1511.41), but higher than the EC's upper limit (1200). Furthermore, Gupta *et al.* (2025) [11] further reinforce the present findings by showing, through a comparative assessment of routine CT dose indices across two tertiary care hospitals in Delhi NCR, that inter-institutional variation in DLP and CTDI persists in clinical practice, highlighting the importance of ongoing protocol review, dose monitoring, and optimization to improve radiation safety without compromising diagnostic utility.

## Conclusion:

Radiation doses from two Computed Tomography scanners show that the Siemens Somatom scanner consistently delivers lower CTDIvol and Dose Length Product values, staying within or below diagnostic reference levels (DRLs). However, the GE scanner produces high doses, particularly in chest and head CTs, often exceeding DRLs. These findings emphasize the need for protocol optimization, scanner-specific calibration and periodic dose audits to ensure patient safety and adherence to ALARA principles.
